# Complement C4, C4A and C4a – What they do and how they differ

**DOI:** 10.1016/j.bbih.2024.100809

**Published:** 2024-06-11

**Authors:** Meike Heurich, Melanie Föcking, David Cotter

**Affiliations:** a School of Pharmacy and Pharmaceutical Sciences, College of Biomedical and Life Sciences, Cardiff University, United Kingdom; b Department of Psychiatry, Royal College of Surgeons in Ireland, University of Medicine and Health Sciences, Dublin, Ireland

**Keywords:** Psychosis, Schizophrenia, Complement system, Complement C4, C4A

Schizophrenia has been linked to the immune system, in particular the major histocompatibility complex class III (MHC), variations in complement component 4 (C4), and a risk association with increased expression of the C4A isotype (Sekar et al., 2016). While the functional role of the C4A protein in pathophysiology remains largely unknown, a more recent study showed that overexpression of C4A promotes excessive synaptic pruning (Yilmaz et al., 2021).

The complement system is part of the innate, non-cellular immune system, important for pathogen clearance, including opsonization and clearance of microorganisms, apoptotic debris and antibody-antigen complexes or immune complexes (Merle et al., 2015).

Several studies in the psychosis and schizophrenia field have now measured complement C4 or C4 fragments in biological samples, such as blood plasma or cerebrospinal fluid (CSF) (Gangadin et al., 2023; Gracias et al., 2022; Hatzimanolis et al., 2022; Kalinowski et al., 2021; Liu et al., 2022; Susai et al., 2023). Some measured C4A protein (Gangadin et al., 2023; Gracias et al., 2022) and others quantified C4a (Susai et al., 2023; Kalinowski et al., 2021). While C4a can be readily quantified, with various commercial C4a ELISA (Enzyme-linked immunosorbent assays) available, we note that some use the C4a and C4A/B nomenclature interchangeably.

It is noteworthy that C4A and C4a, while both complement components and valuable complement markers, are not the same molecule. Thus, interpretation of the respective results, while valid, needs to be specific to each molecule's role in complement activation, especially when put into context with C4 expression levels. This brief overview seeks to clarify the difference between C4A and C4a and to aid in the interpretation of the C4A and C4a changes observed in psychosis.

The complement system comprises three pathways activated by distinct mechanisms: classical (CP), lectin (LP) and alternative pathway (AP). C4 is involved in the classical and lectin complement pathways leading to complement activation, resulting in the downstream generation of inflammatory and pore-forming molecules (Merle et al., 2015).

## 1 Complement C4 activation

**Fig. 1:**
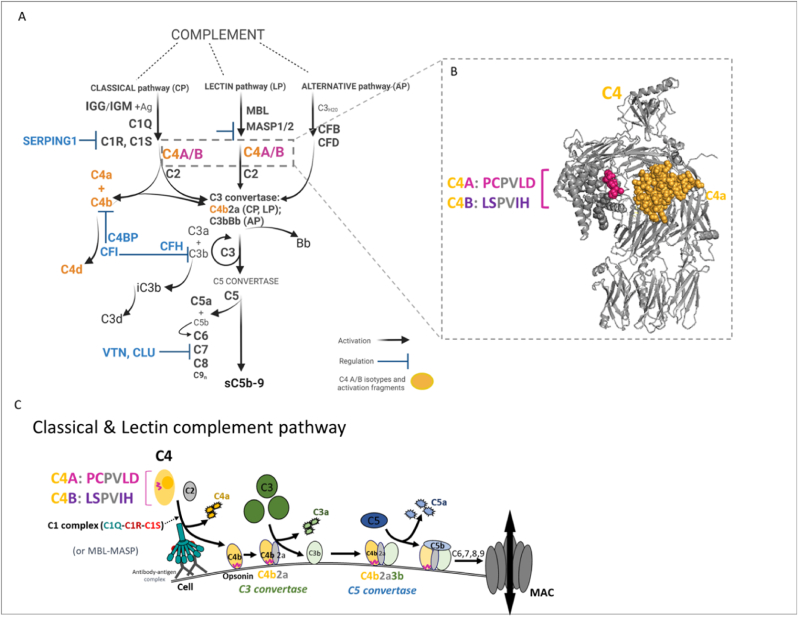
**A.** Overview of the complement system. The complement system comprises three pathways activated by distinct mechanisms: classical (CP), lectin (LP) and alternative pathway (AP). The classical complement pathway is triggered by antibody-antigen complexes, and the lectin pathway by carbohydrates on the surface of pathogens. In the classical pathway, antibody-antigen complexes activate the C1 complex (namely C1q-C1r_2_-C1s_2_), where C4 is cleaved by the enzyme C1s into C4a and C4b. C4b can be further cleaved into C4d by factor I (CFI) and C4-binding protein (C4bp). The C1 complex is regulated by C1 inhibitor (SERPING1). In the presence of bacterial carbohydrates, the lectin pathway is activated through the MBL-MASP complex, with C4 being cleaved by the enzyme MASP2. C1s or MASP2 cleave the C4 and C2 proteins to form the C3 convertase (C4b2a) enzyme leading to activation of C3 (Merle et al., 2015). **B** Complement C4 protein is expressed as two isotypes, C4A and C4B. The difference lies in four isotype-specific amino acid substitutions at positions amino acid positions 1120–1025 (C4A: ^1120^PCPVLD^1125^; C4B: ^1120^LSPVIH^1125^). The C4 protein structure (pdb 5JPN) was visualised using PyMOL (http://www.pymol.org/pymol) and is shown in grey; isotype-specific amino acids are highlighted in pink and the C4a fragment is shown in yellow. **C**C4 is cleaved into C4a and C4b. C4a function as an anaphylatoxin remains under debate, but nascent C4b can be covalently deposited on surfaces and functions as an opsonin. Upon C4 cleavage into C4a and C4b, the isotypic region remains with C4b (highlighted in pink). Thus, the C4A protein variant is cleaved into C4a and C4bA, while C4B is cleaved into C4a and C4bB. Either C4b binds C2 and forms the CP/LP C3 convertase (C4b2a), which is then able to cleave C3 into C3a (anaphylatoxin) and opsonin C3b. C4b2aC3b form the C5 convertase, which cleaves C5 into C5a (anaphylatoxin) and C5b, with subsequent binding of C6-7-8-9, and formation of the pore-forming membrane attack complex (MAC, C5b-9). Figure “Created with BioRender.com”. (For interpretation of the references to colour in this figure legend, the reader is referred to the Web version of this article.)

The classical complement pathway is triggered by antibody-antigen complexes, and the lectin pathway by carbohydrates on the surface of pathogens. Triggering these pathways leads to the activation of serine proteases that cleave, or activate, complement component C4. In the classical pathway, antibody-antigen complexes activate the C1 complex (namely C1q-C1r_2_-C1s_2_), where C4 is cleaved by the enzyme C1s. In the presence of microbial carbohydrates, the lectin pathway is activated through the MBL-MASP complex, with C4 being cleaved by the enzyme MASP2. These enzymes (C1s, or MASP2) cleave the C4 molecule into two fragments: C4a (small fragment) and C4b (large fragment) and are regulated by C1 Inhibitor (SERPING1). During this process, nascent C4b can bind covalently to surfaces and functions as an opsonin, which is to deposit on cell surfaces and make them more susceptible to phagocytic uptake by immune cells, including microglia in the brain. C4b is also a building block for the C3 convertase (C4b2a), which then goes on to cleave complement component C3 into C3a and C3b, the latter also being an opsonin. C4b is also a building block for the C5 convertase (C4b2aC3b), which cleaves complement component C5 into C5a and C5b; this subsequently leads to formation of the membrane attack complex (MAC, C5b-9) (Merle et al., 2015), as summarised in Fig. 1A.

## 2 Complement C4, C4A and C4a

Complement C4 protein is expressed as two isotypes, C4A and C4B, which are encoded by the C4 genes (*C4A* or *C4B* gene), and share >99% sequence identities. The difference lies in four isotype-specific amino acid substitutions at amino acid positions 1120–1025 (C4A: ^1120^**PC**PV**LD**^1125^; C4B: ^1120^**LS**PV**IH**^1125^). The A/B reflect the more Acidic (C4A) or Basic (C4B) electrophoretic mobilities at alkaline pH (Isenman 2018).

Upon complement activation, C4 (either C4A or C4B) is enzymatically cleaved into C4a and C4b. Thus, C4a is a structural fragment within the C4 structure, in both C4A and C4B. Fig. 1B shows the molecular structure of C4 (pdb 5JPN), with the isotypic peptide that denotes it as C4A shown in magenta and the C4a fragment shown in orange.

Upon C4 cleavage into C4a and C4b, the isotypic region remains with C4b, as highlighted in Fig. 1C. Thus, the C4A protein variant is cleaved into C4a and C4bA, while C4B is cleaved into C4a and C4bB. For each C4A/B isotype, the C4b-to-surface binding has shown preferential molecular binding partners, amino groups for C4A and hydroxyl groups for C4B, which may explain some functional differences between the isotypes (Isenman 2018). While the structure-function relationship of C4A in the context of schizophrenia remains unknown, there is a risk association with higher C4A levels (Sekar et al., 2016). Noting that the isotypic region is contained in the opsonin C4bA or C4bB, this suggests a functional role relevant to opsonization or surface-binding ability. Nevertheless, the C4a fragment can be measured and readily quantified in blood and is a proportional measure of total C4 activation. Altered C4a levels have been observed in a number of diseases, including neurological disorders (Nijs et al., 2010). Several psychosis and schizophrenia studies have measured C4a (Susai et al., 2023; Kalinowski et al., 2021; Liu et al., 2022). Increased C4a levels can be a useful potential biomarker to indicate increased C4 activation. However, while increased C4A variant levels could potentially affect C4a generation by contributing to increased total C4 levels, the C4a molecule is strictly speaking isotype independent.

In summary, C4A is an isotypic variant of the C4 molecule, while C4a is a protein fragment that is released from the C4 molecule when C4 is proteolytically activated. The isotypic region that is designated as A or B is present in C4 and remains in the enzymatically generated C4b opsonin, thus C4A gives rise to C4bA. On the molecular level, C4a derived from either C4A and C4B is indistinguishable and reflects on total C4 activation. In contrast, the C4 isotypes differ in their amino acid sequence, which affects their preference of surface-binding partners and thus suggests differences in function. To relate changes in either C4a or C4A to pathophysiological mechanisms, further work is required to accurately quantify total C4, the C4A/B variants and C4a.

## CRediT authorship contribution statement

**Meike Heurich:** Conceptualization, Visualization, Writing – original draft, Writing – review & editing. **Melanie Föcking:** Writing – original draft, Writing – review & editing. **David Cotter:** Writing – original draft, Writing – review & editing.

## Declaration of competing interest

The authors declare that they have no known competing financial interests or personal relationships that could have appeared to influence the work reported in this paper.

## Data Availability

No data was used for the research described in the article.
